# Effects of Cold-Light Bleaching on Enamel Surface and Adhesion of *Streptococcus mutans*

**DOI:** 10.1155/2021/3766641

**Published:** 2021-08-21

**Authors:** Bo Zhang, Sibei Huo, Shiyu Liu, Ling Zou, Lei Cheng, Xuedong Zhou, Mingyun Li

**Affiliations:** ^1^State Key Laboratory of Oral Diseases & National Clinical Research Center for Oral Diseases, West China Hospital of Stomatology, Sichuan University, Chengdu, China; ^2^Department of Orthodontics, West China Hospital of Stomatology, Sichuan University, Chengdu, China; ^3^Department of Stomatology, Ministry of Education Key Laboratory of Child Development and Disorders, National Clinical Research Center for Child Health and Disorders (Chongqing), China International Science and Technology Cooperation Base of Child Development and Critical Disorders, Children's Hospital of Chongqing Medical University, Chongqing Key Laboratory of Pediatrics, Chongqing, China; ^4^Department of Operative Dentistry and Endodontics, West China Hospital of Stomatology, Sichuan University, Chengdu, China

## Abstract

Tooth bleaching is becoming increasingly popular among patients with tooth staining, but the safety of bleaching agents on tooth structure has been questioned. Primarily thriving on the biofilm formation on enamel surface, *Streptococcus mutans* has been recognized as a major cariogenic bacterial species. The present study is aimed at investigating how cold-light bleaching would change enamel roughness and adhesion of *Streptococcus mutans*. Human premolars were divided into 72 enamel slices and allocated into 3 groups: (1) control, (2) cold-light bleaching with 35% hydrogen peroxide (Beyond^™^), and (3) 35% hydrogen peroxide (Beyond^™^) alone. Biofilms of *Streptococcus mutans* were cultivated on enamel slices in 5% CO_2_ (*v*/*v*) at 37°C for 1 day or 3 days. Enamel surfaces and biofilms were observed using scanning electron microscope (SEM). Atomic force microscopy (AFM) was applied to quantify the roughness of enamel surface, and the amounts of biofilms were measured by optical density of scattered biofilm and confocal laser scanning microscopy (CLSM). Cold-light bleaching significantly increased (*p* < 0.05) surface roughness of enamel compared to controls, but significantly inhibited (*p* < 0.05) adhesion of *Streptococcus mutans* on enamel in the bacterial cultures of both 1 day and 3 days. In conclusion, cold-light bleaching could roughen enamel surface but inhibit *Streptococcus mutans* adhesion at the preliminary stage after the bleaching treatment.

## 1. Introduction

Tooth bleaching has enjoyed great popularity among patients suffering from intrinsic and extrinsic tooth staining. It can efficiently improve the shade of dental fluorosis, tetracycline pigmentation teeth, and pulpless tooth [1]. Hydrogen peroxide (HP) or carbamide peroxide (CP) is the main component of the majority of bleaching products on the market. When applied on teeth, HP releases HO_2_^−^ and OH [2], which diffuse through enamel and dentin, react with pigments or chromophores, and also change the reflection of tooth surface [3]. CP, which is rapidly decomposed of HP and urea after applied on teeth, shares similar bleaching mechanism with HP. Besides, urea from CP can degrade organic matrix in enamel, facilitating the diffusion of bleaching agents through enamel to reach the dentino-enamel junction [4, 5]. Application of lights can effectively assist the bleaching procedure, and cold-light bleaching has been proved effective to whiten teeth [6]. The efficacy is based on the application of high-concentrated bleaching agents combined with the activation of special blue light (wavelength between 480 nm and 520 nm). The cold-light lamp is equipped with filter to exclude the harmful infrared (wavelength *λ* > 750 nm) and ultraviolet (*λ* < 380 nm) to reduce the risk of thermal pulp damage and possible side effects on living cells. In the case of cold-light bleaching, light can activate peroxide to promote the chemical redox reactions in the bleaching process [6].

To investigate the negative effect of bleaching treatment and improve bleaching products, numerous *in vivo* and *in vitro* studies have focused on the changes of morphology and microecology in oral cavity after bleaching treatment. Detrimental alterations in enamel have been reported, including the increase of surface roughness and the decrease of microhardness [5–9]. However, other studies found no significant changes in those aspects [10, 11]. Either is there any consensus on the effect of tooth bleaching on bacteria adhesion to enamel. Given to the increasing popularity of cold-light bleaching, the knowledge of the potential side effects is necessary for both applicants and patients. To our knowledge, nevertheless, only a limited number of studies have reported the effect of cold-light bleaching on enamel surface and adhesion of oral bacteria on enamel at the same time.

As a group of highly adherent bacteria, *Streptococcus mutans* (*S. mutans*) are one of the primary pathogens in the development of dental caries. Producing acids and bacteriocin, they are highly tolerant to acid and possess high-affinity systems for the assimilation of various carbohydrate sources. Thus, this study is aimed at investigating the effect of cold-light bleaching on enamel surface and adhesion of *S. mutans* on enamel. The null hypotheses are as follows: (1) cold-light bleaching has no effect on enamel surface and (2) it has no effect on bacteria adhesion.

## 2. Materials and Methods

### 2.1. Tooth Selection

Twenty-four maxillary premolars extracted for orthodontic purpose, with no caries lesions, enamel hypoplasia, cracks, or other defects on axle enamel surfaces, were collected. Volunteers all signed informed consent, and the study gained approbation of West China Hospital of Stomatology Institutional Review Board (WCHSIRB-ST-2015-128). The collected teeth were rinsed under high-pressure water for 10 min to remove the materia alba [6]. Then, they were stored in 2% formaldehyde solution at 4°C till following treatment [12].

### 2.2. Sample Treatment

Previously described protocol was applied with minor modifications [12]. Every tooth was sectioned into mesiobuccal, distobuccal, and lingual specimens after the removal of root, and enamel surfaces were flattened and polished serially with wet 800-, 1500-, 2000-, 4000-, 5000-grit silicon carbide abrasive papers (Struers, Cleveland, USA). Then, a 3 × 3 × 2 mm enamel slice was cut from every flatted enamel sample using water-cooled saw (Isomet, Buehler, Lake Bluff, IL, USA). Slices were washed in running deionized water for 10 min and dried with compressed air for 5 s before the bleaching treatment.

To control interfering factors from individual variations among every tooth, we randomly allocated three specimens from one tooth into the three groups: (1) control, (2) cold-light bleaching, and (3) 35% hydrogen peroxide (Beyond^™^) alone.

### 2.3. Bleaching Procedure

The cold-light bleaching was processed according to the manufacturer's instructions. 35% HP gel (BEYONS II, Beyond^™^, Beijing, China) was applied on the enamel surface in the treatment group, and the thickness of gel was approximately 2 mm. The bleaching process lasted for 8 min with the cold-light lamp (BY-0398, Beyond^™^, Beijing, China) vertically 1 cm above enamel slices (power density = 500 mW/cm^2^, energy density = 240 J/cm^2^). The bleaching procedure was repeated twice, and the gel was removed during the intervals. The control group was mock-treated by 0.9% saline (*w*/*v*) along with cold-light bleaching for the same time as the bleached group. After bleached, all enamel specimens were washed in running deionized water again for 1 min and then sterilized [6].

### 2.4. Bacterial Strains and Growth Conditions

*Streptococcus mutans* UA159 (ATCC 700610) were maintained in Brain Heart Infusion (BHI) broth. Bacteria were incubated in BHI with 1% (*w*/*v*) sucrose (BHIS) for biofilm formation. The biofilms were incubated in the condition of 5% CO_2_ (*v*/*v*) at 37°C without agitation.

### 2.5. Growth of Biofilm

A protocol of biofilm formation described previously was conducted with some modifications [13]. All enamel specimens were immersed in filter-sterilized saliva (100 ml) from healthy volunteers at 37°C for 2 h to form acquired pellicle [14]. For the biofilm formation, the bacteria were grown in BHI overnight and then diluted by fresh BHI till the optical density equaled 0.2 at 600 nm. 100 *μ*l prepared planktonic bacteria mixed with 900 *μ*l fresh BHI with 1% (*w*/*v*) sucrose (BHIS) was added to each well of 48-well tissue culture plate, in which the specimens were placed with the enamel surface uppermost. The plate was placed in 5% CO_2_ (*v*/*v*) at 37°C, and specimens were transferred into new wells containing fresh BHIS every 24 h. Biofilms on enamel specimens were collected after cultured for 1 day (*n* = 3 for each group) or 3 days (*n* = 3 for each group). Samples were rinsed by sterilized water to eliminate planktonic bacteria, and biofilm on them was removed by cell scrapers and then suspended in 100 *μ*l saline (0.9%, *w*/*v*) in tubes. Biofilms were sonicated for 10 min to separate cells by an ultrasonifier (output control at 8 and duty cycle of 70; Branson Sonifier 450, Fisher Scientific, USA). The turbidity was obtained by optical density (OD) at 595 nm using a microplate reader (SpectraMax 190, Molecular Devices Inc., Sunnyvale, CA) to quantify the amounts of biofilms on enamel.

### 2.6. Scanning Electron Microscope (SEM) Analysis

The sterile specimens were coated by gold and sent to perform SEM (*n* = 3 for each group). For the scanning of biofilm, the specimens adhered by *S. mutans* were fixed with 2.5% glutaraldehyde at 4°C overnight, dehydrated in series concentration of ethanol ranging from 30% to 100%, and sputter-coated with gold [14]. Specimens were scanned at ×20,000 and ×10,000 magnifications.

### 2.7. Atomic Force Microscopy (AFM) Analysis

The AFM test was performed according to previous descriptions [15, 16] with some modifications (*n* = 3 for each group). For surface roughness, the images of morphology and values of roughness average (Ra, nm) were observed by SPM-9600 AFM system (Shimadzu, Kyoto, Japan) in the tapping mode with a silicon nitride tip of NSG11 (NT-MDT, Moscow, Russia) under ambient circumstances. Each specimen was scanned at three randomly selected sites covering an area of 10 × 10 *μ*m at 1 Hz scanning rate. Adhesion forces of the enamel surface were measured in the contact mode with a tipless cantilever. Seventy force-distance curves were attained at seven random regions for each slice at a scanning rate of 0.5 Hz, ramp size of 18 *μ*m, and trigger force of 5 nN. A built-in software within the STM9700 system was applied to calculate adhesion forces from the curves.

### 2.8. Confocal Laser Scanning Microscopy (CLSM)

Enamel specimens were placed in 48-well plates with 100 *μ*l prepared planktonic bacteria (OD = 0.2 at 600 nm), 900 *μ*l BHIS, and Alexa Fluor 647 (10,000 MW; Molecular Probes Inc., USA) to label formed exopolysaccharide (EPS) as previously described [17]. The plates were incubated in 5% CO_2_ (*v*/*v*) at 37°C in the dark for 1 day (*n* = 3 for each group) or 3 days (*n* = 3 for each group), during which specimens with biofilm were transferred into new wells with fresh BHIS and Alexa Fluor 647 every 24 h. After incubation, the specimens were rinsed by 0.9% saline (*w*/*v*) to remove the planktonic cells and then dried with a sterile filter paper. SYTO 9 nucleic acid stain (Molecular Probes Inc., USA) was applied to label *S. mutans* for 15 min. Specimens were washed and dried again. The whole process was completed in the dark, and the stained specimens were glued on glass slides for laser scanning confocal microscopy (Leica TCS SP2, Leica Microsystems, Wetzlar, Germany) which was equipped with a 60x oil immersion objective lens. 485 and 650 nm were used, respectively, as the absorption maxima wavelength for the nucleic acid stain and the EPS dye, and 498 nm was used as the emission maxima wavelength. At least three randomly selected positions of each specimen were captured. Images were taken from the bottom of the biofilm, section by section to the top layer of the biofilm instructed by previous study [18]. 3D images of the biofilm and the ratio of EPS/bacteria were obtained by IMARIS 7.0.0 (Bitplane, Zürich, Switzerland).

### 2.9. Statistical Analysis

Each experiment was repeated at least three times independently. All data were analyzed by SPSS 16.0. We set the level of significance to be 0.05. The Shapiro-Wilk test was used to test the distribution of data first. The independent *t*-test was used to compare the difference between groups for turbidity of scattered biofilm solution, while adhesion forces were analyzed by nonparametric analysis (Kruskal-Wallis test).

## 3. Results

### 3.1. The Roughness of Enamel Surfaces Was Increased after Bleaching

**Figure 1:**
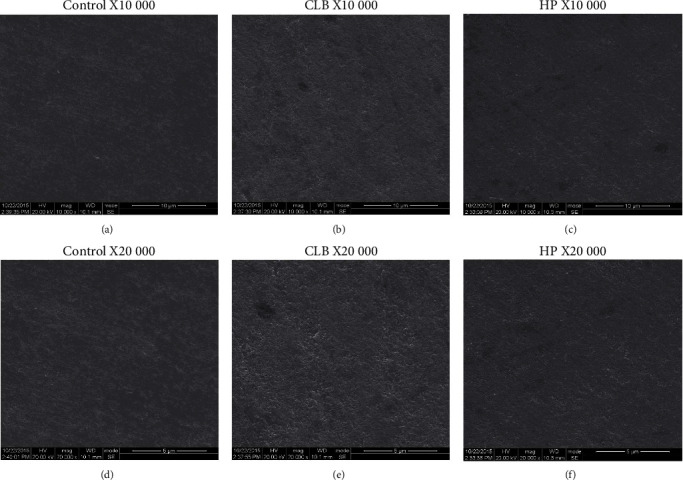
Scanning electron microscope (SEM) images of enamel surface. (a) Control specimen (×10,000 original magnification). (b) Cold-light bleached (CLB) specimen (×10,000 original magnification). (c) Hydrogen peroxide bleached (HP) specimen (×10,000 original magnification). (d) Control specimen (×20,000 original magnification). (e) CLB specimen (×20,000 original magnification). (f) HP specimen (×20,000 original magnification).

**Figure 2:**
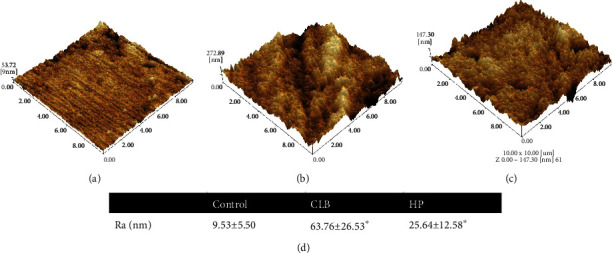
Atomic force microscopy (AFM) images of enamel surface. (a) Three-dimensional image of unbleached specimen. (b) 3D image of cold-light bleached specimen. (c) 3D image of hydrogen peroxide bleached specimen. (d) Roughness average (Ra, nm) values of specimens.

**Figure 3:**
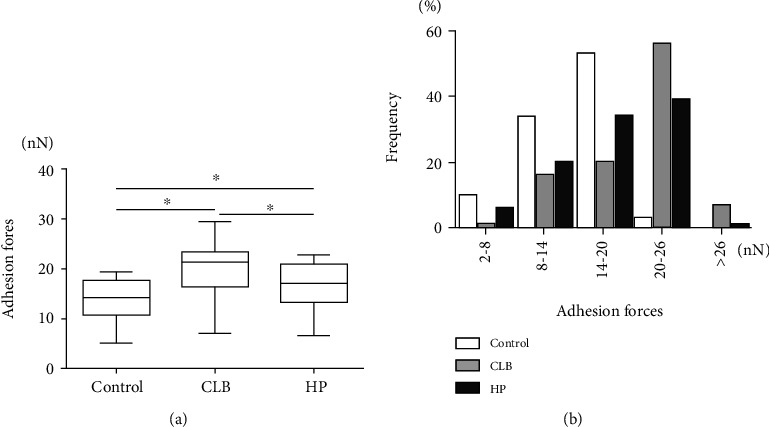
Adhesion forces analyzed from AFM. (a) Adhesion forces presented as mean with 5%-95% percentile (^∗^*p* < 0.05). (b) Distribution of adhesion forces of specimens.

We combined the analyses of SEM and AFM to investigate morphological alterations of enamel surfaces caused by bleaching procedure. In the cold-light bleaching (CLB) group and 35% hydrogen peroxide (HP) group, enamel surface morphology became rougher with more pittings in images of SEM (Figure 1) and 3D images of AFM (Figures 2(a)–2(c)). Clear spherical enamel crystal structures could be observed, suggesting the demineralization of tooth enamel surface by bleaching treatment (Figure 1). The changes were more evident in the CLB group than the HP group (Figure 1). The quantitative results of Ra (nm) analyzed from AFM scanning presented higher Ra (nm) value in CLB and HP specimens than control specimens (Figure 2(c)). Besides, the adhesion forces of enamel surface were significantly higher (*p* < 0.05) in the CLB and HP groups than in the control group (Figures 3(a) and 3(b)).

### 3.2. The Adhesion of *S. mutans* Was Decreased on Bleached Enamel in Turbidity Test

**Figure 4:**
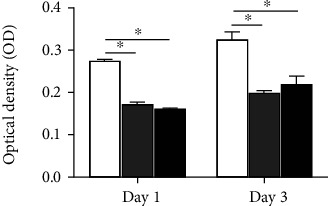
Values of OD of scattered biofilms incubated for 1 day or 3 days on specimens, presented as the mean ± deviation (^∗^*p* < 0.05).

Next, we tested *S. mutans* adhesion on enamel surfaces via turbidity test. The solution of scattered biofilm cultured for 3 days had significantly higher (*p* < 0.05) OD values comparing to that incubated for 1 day (Figure 4). The OD values of scattered biofilm solution collected from the CLB and HP enamels were significantly lower (*p* < 0.05) than those from the control group, which implicated the possible bacteria inhibitory effect of bleaching procedure (Figure 4). There was no significant difference between the CLB group and HP group (*p* > 0.05).

### 3.3. The Thickness of Biofilms Was Decreased on Bleached Enamel

**Figure 5:**
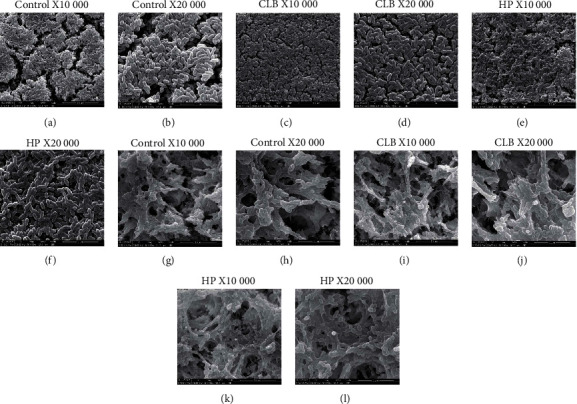
SEM images of biofilms. (a–f) Biofilms incubated for 1 day. (a, c, e) On unbleached, cold-light bleached (CLB), and hydrogen peroxide bleached (HP) enamel (×10,000 original magnification). (b, d, f) On unbleached, CLB, and HP enamel (×20,000 original magnification). (g–l) Biofilms incubated for 3 days. (g, i, k) On unbleached, CLB, and HP enamel (×10,000 original magnification). (h, j, l) On unbleached, CLB, and HP enamel (×20,000 original magnification).

SEM was applied to observe biofilms of *S. mutans* on enamel after incubation for 1 day and 3 days. From SEM images of biofilms incubated for 1 day, thinner *S. mutans* biofilms were formed on CLB and HP specimens (Figures 5(c)–5(f)) than on control ones (Figures 5(a) and 5(b)), indicating that *S. mutans* were less likely to adhere to bleached enamel. After incubation for 3 days, there was no significant visual difference in the amounts of bacteria between the bleached (Figures 5(i)–5(l)) and unbleached groups (Figures 5(g) and 5(h)).

### 3.4. *S. mutans* Were Less Inclined to Adhere on Bleached Enamel from Analysis of CLSM

**Figure 6:**
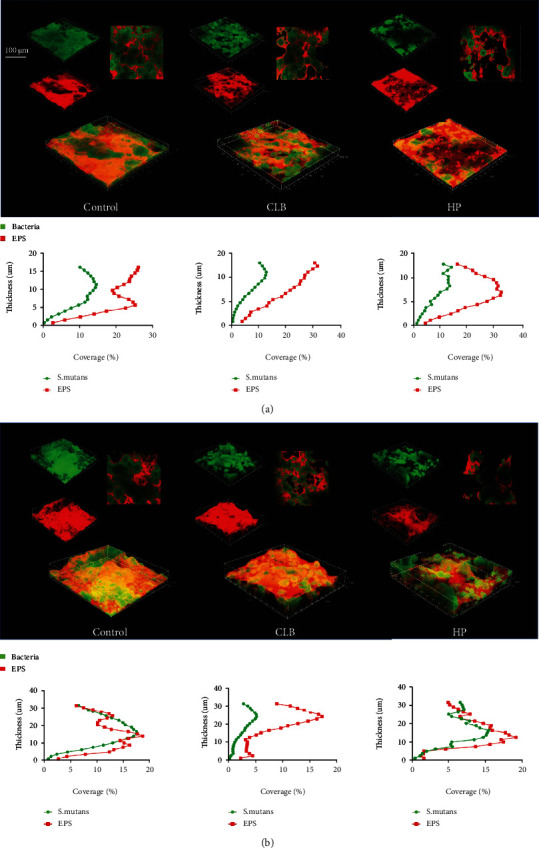
Confocal laser scanning microscopy images of *S. mutans* biofilms. (a) The three-dimensional reconstruction of the *S. mutans* biofilms incubated for 1 day on control and bleached slices and the correspondent distribution of EPS and bacteria of the reconstructed biofilms. (b) The three-dimensional reconstruction of the *S. mutans* biofilms incubated for 3 days on control and bleached slices and the correspondent distribution of EPS and bacteria of the reconstructed biofilms.

Compared to biofilms incubated for 1 day, there was an apparent increase in the thickness of *S. mutans* (green) and EPS (red) in biofilms cultured for 3 days (Figure 6). From 3D reconstruction images (Figure 6), biofilms on CLB and HP specimens were less dense than control ones after incubation for 1 day and 3 days, indicating the comprised adhesion ability of *S. mutans* on CLB and HP enamels.

## 4. Discussion

Up to now, there is no agreement on whether bleaching treatment exerts adverse effects on enamel structure and adhesion of caries-associated microorganisms. One of the proposed mechanisms of tooth bleaching is the agents' effect on changing the reflection of light of the enamel. The increase in surface roughness after tooth whitening may lead to increased reflectance spectra and therefore to improved digital color reading [3, 19, 20]. Moreover, some studies have suggested that demineralization during tooth bleaching contributed to the efficacy of whitening [3, 21, 22], which was supported by a phenomenon of color regression after bleaching procedure associated with increased mineral uptake [23]. Thus, we evaluated the change of enamel surface morphology after cold-light bleaching. Considering the disadvantages of SEM, including the low pressure ambient and acquired gold sputtering which may change natural condition of tested samples [24], we supplemented AFM scanning to provide 3D images of surfaces and more quantitative data to demonstrate the increase of surface roughness after bleaching treatment, especially after cold-light bleaching. The outcome of the morphological alteration of enamel in our study is in accordance with some previous studies. Hosoya et al. concluded that 35% HP could lead to the increase of roughness of enamel according to the test of a noncontact surface roughness shape-measuring apparatus [25]. Besides, rougher enamel surface after cold-light bleaching was reported [6, 26]. It was proposed that demineralization of enamel after bleaching treatment depended on the pH value of agents rather than peroxide *per se* [27]. We observed rougher enamel surfaces in the CLB group than in the HP alone group, which might be explained by the activation of chemical redox reactions by cold light.

In addition, the inhibited biofilm formation of *S. mutans* on both CLB and HP enamels in our study is also consistent with some previous in vitro and in vivo studies. Yuan et al. [28] found the inhibitory effect of cold-light bleaching on the adhesion of mix bacteria in an artificial oral cavity model for 36 h. In an in vitro study, Zheng et al. [13] reported a decrease of *S. mutans*' adhesion to bleached enamel comparing to unbleached one during the first two weeks after cold-light bleaching. A similar change of bacteria adhesion after cold-light bleaching was also reported in an *in vivo* study [29]. Besides, Gursoy et al. found a declined level of plaque index on the third day after treating enamel surface by 35% HP assisted with light [30].

The roughness of enamel surface increased after being bleached, while *S. mutans*' adhesion to bleached enamel decreased. This might be the effect of the residual agents' effect in enamel [31]. The figures of distribution of EPS and bacteria obtained from CLSM also displayed a lower growing speed of bacteria from the bottom of biofilm on bleached specimens, which might indicate the inhibitory effect of residual HP.

However, Ittatirut et al. [14] reported a decrease in enamel surface roughness after being bleached and no significant difference in *S. mutans*' adhesion between bleached and unbleached enamels after incubated for 24 h. Besides, Hosoya et al. [25] reported more *S. mutans* adhering to bleached enamel in the biofilms incubated for 3 days. Indeed, the seemingly contradiction of those studies with ours can probably be explained by the antiseptic effect of bleaching agents itself and its roughening influence on enamel surface. The time it takes to eliminate the residual agents to the minimal inhibitory concentration depends on the types of agents, whitening process, and enamel rinsing procedure after bleaching. Different experimental designs could also have varied impacts on the morphology of enamel, and there is still no agreement on the correlation between the morphology of enamel and adhesion of bacteria.

## 5. Conclusion

Within the limitation of this in vitro study, we concluded that cold-light bleaching could significantly increase enamel surface roughness but inhibit the formation of biofilms of *S. mutans* till 3 days. In order to investigate the change of biofilm adhesion after bleaching treatment, future researches are needed to investigate the formation of biofilms in a relatively longer period. Also, it is necessary to explore the adverse effects of different sorts of bleaching treatments on enamel and bacteria adhesion to improve products and procedures.

## Data Availability

The data that support the findings of the study is available from the corresponding author upon reasonable request.
